# LinearCoFold and LinearCoPartition: linear-time algorithms for secondary structure prediction of interacting RNA molecules

**DOI:** 10.1093/nar/gkad664

**Published:** 2023-08-31

**Authors:** He Zhang, Sizhen Li, Ning Dai, Liang Zhang, David H Mathews, Liang Huang

**Affiliations:** Baidu Research, Sunnyvale, CA, USA; School of Electrical Engineering & Computer Science, Oregon State University, Corvallis, OR, USA; School of Electrical Engineering & Computer Science, Oregon State University, Corvallis, OR, USA; School of Electrical Engineering & Computer Science, Oregon State University, Corvallis, OR, USA; School of Electrical Engineering & Computer Science, Oregon State University, Corvallis, OR, USA; Department of Biochemistry & Biophysics,Rochester, NY 14642, USA; Center for RNA Biology, Rochester, NY 14642, USA; Department of Biostatistics & Computational Biology, University of Rochester Medical Center, Rochester, NY 14642, USA; School of Electrical Engineering & Computer Science, Oregon State University, Corvallis, OR, USA

## Abstract

Many RNAs function through RNA–RNA interactions. Fast and reliable RNA structure prediction with consideration of RNA–RNA interaction is useful, however, existing tools are either too simplistic or too slow. To address this issue, we present LinearCoFold, which approximates the complete minimum free energy structure of two strands in linear time, and LinearCoPartition, which approximates the cofolding partition function and base pairing probabilities in linear time. LinearCoFold and LinearCoPartition are orders of magnitude faster than RNAcofold. For example, on a sequence pair with combined length of 26,190 *nt*, LinearCoFold is 86.8× faster than RNAcofold MFE mode, and LinearCoPartition is 642.3× faster than RNAcofold partition function mode. Surprisingly, LinearCoFold and LinearCoPartition’s predictions have higher PPV and sensitivity of intermolecular base pairs. Furthermore, we apply LinearCoFold to predict the RNA–RNA interaction between SARS-CoV-2 genomic RNA (gRNA) and human U4 small nuclear RNA (snRNA), which has been experimentally studied, and observe that LinearCoFold’s prediction correlates better with the wet lab results than RNAcofold’s.

## Graphical Abstract

**Graphical Abstract:**
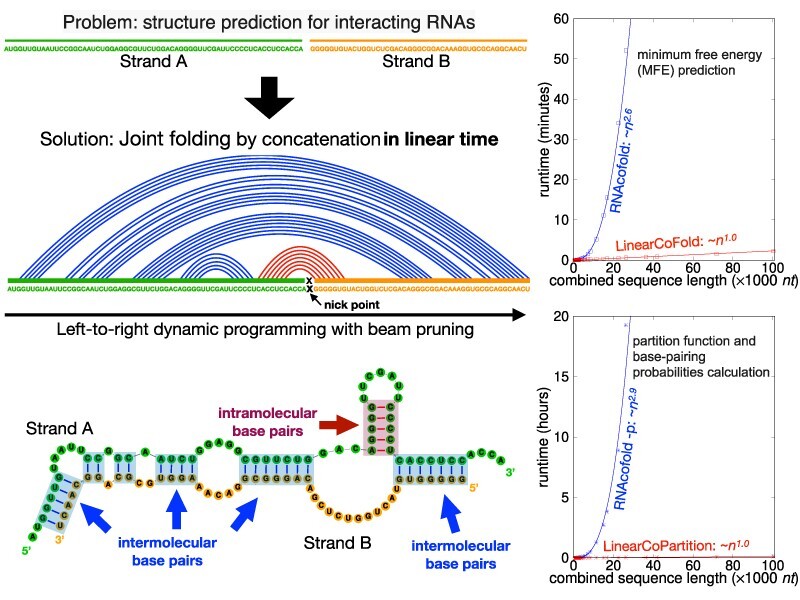

## INTRODUCTION

**Figure 1.**
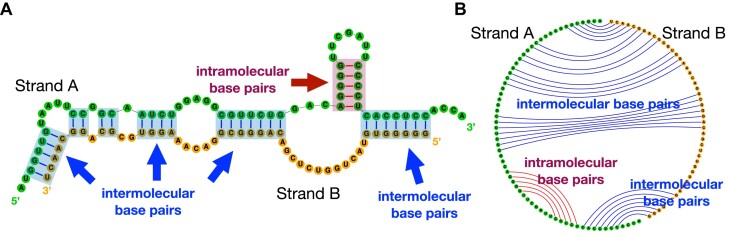
Two RNA strands can form RNA–RNA complexes through intermolecular base pairs. (**A**) The secondary structure of the split tmRNA from *D. aromatica*; two strands are in green and orange, respectively. The intramolecular base pairs are in red, and intermolecular ones are in blue. (**B**) The corresponding circular plot of structure in (A).

RNA strands can interact via intermolecular base pairing and form RNA–RNA complexes. In nature, many RNAs function through these RNA–RNA interactions (Table 1). For instance, it is well-known that microRNA (miRNA) binds with messenger RNA (mRNA) to mediate mRNA destabilization (1) and cleavage (2). Some longer ncRNAs, such as small RNA (sRNA), small nuclear RNA (snRNA) and small nucleolar RNA (snoRNA), involve in RNA–RNA interactions for splicing regulation (3,4) and chemical modifications (5). A small clade of tmRNAs have a two-piece form (i.e., split tmRNA) and form complexes via intermolecular base pairs (see Figure 1). On the other hand, human designed RNAs that bind specifically to the target RNAs are used for diagnostics and treatments. Therapeutic small interfering RNAs (siRNA) trigger RNA interference (RNAi) through siRNA–mRNA interaction (6–8); antisense oligonucleotides (ASOs) bind to target RNA to suppress unwanted gene expression or to regulate splicing (9–11); and CRISPR/Cas-13 guide RNAs (gRNA) induce specific RNA editing by initially binding to the target region (12–14). Fast and reliable secondary structure prediction of interacting RNA molecules is desired to further understand these biological processes and better design diagnostic and therapeutic RNA drugs.

**Table 1.** Interacting RNA molecules are widely distributed in nature, and are involved in multiple biological processes

RNA–RNA interaction	Function
siRNA–mRNA	mRNA degradation
miRNA mRNA	mRNA cleavage, destabilization and down-regulation
sRNA–mRNA	mRNA silencing
gRNA–mRNA	mRNA editing
snRNA–mRNA	RNA splicing and regulation
snoRNA–rRNA	rRNA modification
split tmRNA	rescue of stalled ribosomes; degradation of defective mRNA

**Figure 2.**
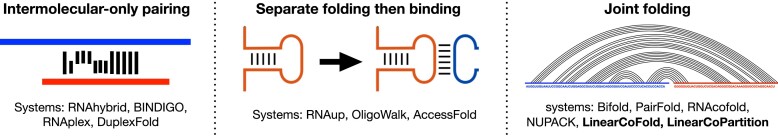
Three categories of the systems for predicting the RNA–RNA interaction and structures.

Some existing systems are used to predict RNA–RNA interaction and structures. We categorize these systems into three categories, and summarize them in Figure 2 and Table 2. The first category, named ‘intermolecular-only pairing’, scans along the target RNA and predicts both the binding site and structure of the intermolecular hybridization. RNAhybrid (15), BINDIGO (16), RNAplex (17) and DuplexFold (18) are four examples of this category. These systems are fast, but they are less informative and less accurate due to only predicting the intermolecular base pairs but omitting the intramolecular ones (19,20). To address this, cascaded systems are proposed, and we group them to the category of ‘separate folding then binding’. As an example of these systems, RNAup (21) firstly calculates the accessibility of windows of interest, then computes the binding energy reward of each window for a given oligo, and finally combines the target region’s accessibility and binding reward together to obtain binding affinity. Similarly, OligoWalk (22) considers self-structures in the equilibrium and then predicts the free energy changes of the hybridization of two sequences. AccessFold (23) also uses such a two-step strategy but adopts a pseudo-energy for the cost of making the target region accessible. The drawback of the systems in this category is the slowness: the first step of these cascaded systems employ an *O*(*n*^3^) algorithm to compute the structure of the target sequence (*n* is the sequence length), resulting in a substantial slow down compared to RNAhybrid and RNAplex.

**Table 2.** An overview of some RNA–RNA interaction prediction tools (all predict intermolecular base pairs)

Category	System	Fold short sequence?	Fold long sequence?	Pseudoknotted pairs?	MFE or partition	Computational complexity
	**RNAhybrid** (15)				MFE	*O*(*nm*)
**Intermolecular-only**	**BINDIGO** (16)				MFE	*O*(*nm*)
**Pairing**	**RNAplex** (17)				MFE	*O*(*nm*)
	**DuplexFold** (18)				MFE	*O*(*nm*)
**Separate folding**	**RNAup** (21)		$\checkmark$	$\checkmark$	partition	*O*(*n*^3^$ {w}$ + *nw*^5^)
**Then binding**	**OligoWalk** (22)	$\checkmark$	$\checkmark$	$\checkmark$	both	*O*(*n*^3^ + *m*^3^ + *nm*)
**(cascaded)**	**AccessFold** (23)	$\checkmark$	$\checkmark$	$\checkmark$	both	*O*(*n*^3^ + *m*^3^ + *nm*)
**Joint folding**	**bifold** (22)	$\checkmark$	$\checkmark$		MFE	*O*((*n* + *m*)^3^)
	**RNAcofold** (36)	$\checkmark$	$\checkmark$		both	*O*((*n* + *m*)^3^)
	**PairFold** (25)	$\checkmark$	$\checkmark$		both	*O*((*n* + *m*)^3^)
	**NUPACK** (26,27)	$\checkmark$	$\checkmark$	$\checkmark$	both	*O*((*n* + *m*)^5^)
	**LinearCoFold**	$\checkmark$	$\checkmark$		MFE	*O*((*n* + *m*)*b*log*b*)
	**LinearCoPartition**	$\checkmark$	$\checkmark$		partition	*O*((*n* + *m*)*b*^2^)

In the computational complexity column, we denote *n* and *m* as the lengths of the two input sequences, *w* as the binding window size, and *b* as the beam size in our LinearCoFold and LinearCoPartition. Note that *w* and *b* are constants; by default, *w* is 25 in RNAup, and *b* is 100 in our algorithms. PairFold and NUPACK are the tools that can do multiple sequence folding, i.e., allowing more than two input sequences. Our LinearCoFold and LinearCoPartition achieve linear runtime for approximate MFE and partition function with the consideration of both inter- and intramolecular base pairs.

The first two categories discussed above compute the binding affinity and predict the binding region, but they are not able to simultaneously fold the competing intra- and intermolecular base pairs, nor to predict the complete binding conformation of two interacting sequences, which, however, are desired in many cases. Figure 1 illustrates the secondary structure in the region of interaction of the split tmRNA from *D. aromatica* (23), showing that both intra- and intermolecular base pairs exist in the binding region. To predict the *joint structure*, the third category of tools, including bifold (22), Vienna RNAcofold (24), PairFold (25) and NUPACK (26,27), were developed. The basic framework of these tools is to concatenate two input sequences as a single sequence, and predict the whole secondary structure of the concatenated sequence based on the classical dynamic programming algorithms. Though there are some differences in the implementations, all of these systems have (at least) *O*((*n* + *m*)^3^) time complexity and *O*((*n* + *m*)^2^) space complexity (NUPACK predicts pseudoknotted structure in *O*((*n* + *m*)^5^) time and *O*((*n* + *m*)^4^) space), where *n* and *m* are the lengths of the two strands. Due to the cubic or worse time complexity, such traditional systems are slow or even impossible to run on long sequences (20,28–34), for example, 16S (∼1500 *nt*) and 23S (∼3000 *nt*) ribosomal RNA, viral RNA genomes such as HIV (∼10 000 *nt*), RSV (∼15 000 *nt*), Ebola (∼18 000 *nt*) and SARS-CoV-2 (∼30 000 *nt*), and even longer transcript sequences (35). In addition, the standard cubic-time algorithms have a quadratic space complexity (memory usage), which prevents many tools from scaling to long sequences (esp. on commodity machines). These limitations also prevent them from being applied for genome-wide scanning (19).

To accelerate and scale up the prediction of the joint structure, we propose LinearCoFold and LinearCoPartition, which follow the ‘concatenation’ strategy to simplify two-strand cofolding into classical single-strand folding, and predict both intramolecular and intermolecular interactions. Different from previous cubic runtime algorithms, LinearCoFold and LinearCoPartition adopt a left-to-right dynamic programming and further apply beam pruning heuristics to reduce their runtime to linear-time. Specifically, LinearCoFold predicts the approximate minimum free energy structure of two strands, while LinearCoPartition computes the approximate partition function and base pairing probabilities, and can output assembled structures with downstream algorithms such as maximum expected accuracy (MEA) (37) and ThreshKnot (38). Unlike other *local* cofolding algorithms, LinearCoFold and LinearCoPartition are *global* linear-time algorithms, i.e. they do not impose any limitations on base pairing distance.

We compare the efficiency and scalability of our algorithms to Vienna RNAcofold, and confirm that the runtime and memory usage of LinearCoFold and LinearCoPartition scale linearly against the combined sequence length, while RNAcofold scales cubically in runtime and quadratically in memory usage. LinearCoFold and LinearCoPartition are orders of magnitude faster than RNAcofold. On the longest data point in the benchmark dataset that RNAcofold can run (26 190 *nt*), LinearCoFold is 86.8× faster than RNAcofold MFE mode, and LinearCoPartition is 642.3× faster than RNAcofold partition function mode. Notably, RNAcofold cannot run on any combined sequences longer than 32 767 *nt*, but our LinearCoFold and LinearCoPartition have no limitation of sequence length internally, and can scale up to sequences of length 100 000 *nt* in 2.2 and 6.9 minutes, respectively. With respect to accuracy, LinearCoFold and LinearCoPartition’s predictions are more accurate in sensitivity (the fraction of known pairs correctly predicted) and Positive Predictive Value (PPV; the fraction of predicted pairs that are in the accepted structure). Compared with RNAcofold (MFE mode), the overall PPV and sensitivity of LinearCoFold increase by +4.0% and +11.6%, respectively; compared with RNAcofold + MEA, LinearCoPartition + MEA gains improvement of +2.9% on PPV and +5.7% on sensitivity; compared with RNAcofold + TheshKnot, LinearCoPartition + TheshKnot increases by +1.5% and +5.2% on PPV and sensitivity, respectively. Furthermore, we demonstrate that our predicted interaction correlates better to the wet lab results of the RNA–RNA interaction between SARS-CoV-2 gRNA and human U4 snRNA, showing that our algorithms can be used as a fast and reliable tool in genome-wide studies.

## MATERIALS AND METHODS

### Extend single-strand folding to double-strand folding by concatenation

Both LinearCoFold and LinearCoPartition take two RNA sequences as input, and reduce the two-strand cofolding to the single-strand folding via concatenating two input RNAs. Formally, we denote the two RNA sequences as $\mathbf {x}^a =x_1^a x_2^a...x_n^a$ and $\mathbf {x}^b =x_1^b x_2^b...x_m^b$, where *n* and *m* are the lengths of **x**^*a*^ and **x**^*b*^, respectively. Thus, the new concatenated sequence of length *n* + *m* can be denoted as $\mathbf {x} =x_1 x_2...x_n \ {\smallriptscriptstyle {(\times )}} \ x_{n+1} x_{n+2}...x_{n+m}$, where the nick point ${\smallriptscriptstyle {(\times )}}$ is between nucleotides *x*_*n*_ and *x*_*n* + 1_.

After this transformation, the classical dynamic programming algorithm for single-strand folding (39,40) can be applied to the concatenated sequence. One thermodynamic change that needs to be considered for this extension is that a structure with intermolecular base pairs incurs a stability penalty for intermolecular initiation (41). Formally, in the Nussinov-Jacobson system, we denote the free energy change of the ‘normal’ base pair (*i*, *j*) as ξ(**x**, *i*, *j*), which includes both intramolecular base pairs and non-intermost intermolecular base pairs; we denote the free energy change of the innermost intermolecular base pair (*i*, *j*) as $\xi (\mathbf {x}, i, j) + G^{\circ }_{\text{DuplexInit}}$, where $G^{\circ }_{\text{DuplexInit}}$ is the free energy change of duplex initiation. Besides, the free energy change of the unpaired base *k* is denoted as δ(**x**, *k*). Thus, the free energy change $\Delta G^{\circ }(\mathbf {x}, \mathbf {y})$ of the concatenated sequence **x** and its structure **y** can be decomposed as:


(1) \begin{eqnarray*} \Delta G^{\circ }(\mathbf {x}, \mathbf {y}) = \sum _{k \in {\rm unpaired}(\mathbf {y})} {\delta (\mathbf {x}, k)} \ \ + {\sum _{(i,j) \in {\rm pairs}(\mathbf {y})} \phi (\mathbf {x}, \mathbf {y}, i, j)}\nonumber\\ \end{eqnarray*}

where


(2) \begin{eqnarray*} {\phi (\mathbf {x}, \mathbf {y}, i, j) = \xi (\mathbf {x}, i, j) + \left\lbrace \begin{array}{@{}l@{\quad }l@{}}\Delta G^{\circ }_{\text{DuplexInit}},& \text{if } (i,j) \text{ is the} \\ & \text{innermost intermole-}\\ & \text{cular base pair in } \mathbf {y} \\ & \\ 0, & \text{otherwise} \end{array}\right.} \end{eqnarray*}

Note that if no base pair covers the nick point, i.e. the two strands do not interact with each other, two-strand cofolding is simply single-strand folding of two strands separately.

**Figure 3.**
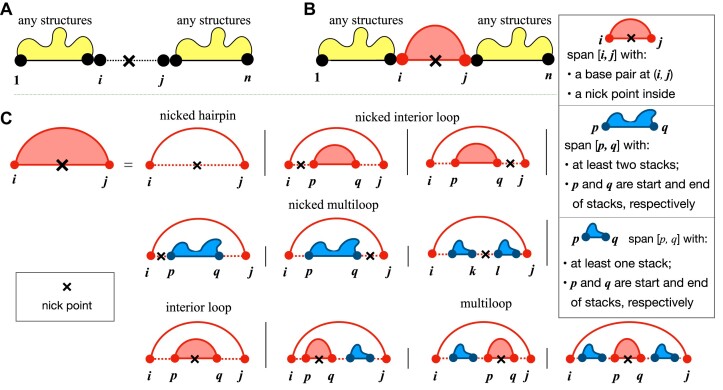
The relative positions of the nick point when concatenating two strands for zuker-style cofolding. (**A**) The nick point is not covered by a base pair, i.e., there is no intermolecular base pairs. (**B**) The nick point is covered by an intermolecular base pair; note that only in this case two stands form a RNA–RNA complex. (**C**) The breakdown cases of the interacting span [*i*, *j*] in (**B**). When the nick point is directly covered by the outside intermolecular base pair (*i*, *j*) (the first and second rows in C), they form no more hairpins, interior loops or multiloops, but exterior loops, so we call them the corresponding ‘nicked’ loops. But when the nick point is covered by a nested base pair (*p*, *q*), they are still normal interior loops and multiloops (the third row in C).

Next, we consider the Zuker system based on the Turner energy model (42–44). More sophisticated than the Nussinov-Jacobson model, the Zuker/Turner scoring system is based on four types of loops: exterior loops, hairpin loops, interior loops (including bulge loops) and multiloops. In Figure 3, we illustrate the relative positions of the nick point in these four types of loops. For the external loop, the nick point can be either covered by a base pair or not (Figure 3A–B). If an intermolecular base pair (*i*, *j*) covers the nick point, the span [*i*, *j*] can be further categorized into three types: nicked hairpin, nicked interior loop and nicked multiloop, based on the type of loops it enclosed (Figure 3C). Specifically, the nicked hairpin loop only requires *i* ≤ *n* < *j*, while the nicked interior loop has an inner loop from position *p* to *q*, and requires either *i* ≤ *n* < *p* or *q* ≤ *n* < *j*; see the first row of Figure 3C for an illustration. The nicked multiloop is more complicated (the second row of Figure 3C):

- the nick point is at the leftmost unpaired region, i.e., it is between *i* and *p* where *p* is the 5’ end of the first multibranch;
- the nick point is at the rightmost unpaired region, i.e. it is between *q* and *j* where *q* is the 3’ end of the last multibranch;
- the nick point is in the middle, i.e. it is between *k* and *l* which are the 3’ end and the 5’ end of two consecutive multibranches, respectively.

Such nicked loops are considered to be exterior loops when calculating their free energy change. Note that the nick point only affects the innermost loop that directly covers it; the loops are still normal interior loops and multiloops in the case that the nick point is covered by another base pair (*p*, *q*) where *i* < *p* < *q* < *j*, shown in the third row of Figure 3C.

The software of LinearCoFold and LinearCoPartition use the Turner energy model, implemented identically to RNAcofold. Compared to the single folding energy parameters, an extra parameter, $G^{\circ }_{\text{DuplexInit}}= 4.1$ kcal/mol, is used for the free energy cost of forming dimers.

### LinearCoFold algorithm

LinearCoFold aims to predict the minimum free energy (MFE) structure of double-strand RNAs in linear runtime without imposing a limit on base pair length. Formally, LinearCoFold finds the MFE structure $\hat{\mathbf {y}}$ among all possible structures $\mathcal {Y}(\mathbf {x})$ under the given energy model **w**:


(3) \begin{equation*} \hat{\mathbf {y}} = {\mathbf {argmin}}_{\mathbf {y} \in \mathcal {Y}(\mathbf {x})}{\Delta G^{\circ }_{\mathbf {w}}(\mathbf {x}, \mathbf {y})} . \end{equation*}

Inspired by LinearFold (28), LinearCoFold adopts a left-to-right dynamic programming (DP), in which we scan and fold the combined sequence from left to right. Supplementary Figure S1 presents the pseudocode of LinearCoFold based on the revised Nussinov-Jacobson energy model. This new DP algorithm is equivalent to the classical algorithm in the sense that they both find the MFE structure in cubic time, however, such left-to-right fashion allows applying beam pruning, which retains the top *b* states with lower folding free energy change at each step *j*. As a result, the time complexity of LinearCoFold is *O*((*n* + *m*)*b*log*b*), where *b* is the beam size and the default value is 100. It is clear in the pseudocode that LinearCoFold does not impose any constraints on base-pairing distance, which is different from the local folding approximation. To extend to two-strands cofolding, LinearCoFold applies the $\Delta G^{\circ }_{\text{DuplexInit}}$ free energy cost for innermost intermolecular pairs as shown in Equation (2).

**Figure 4.**
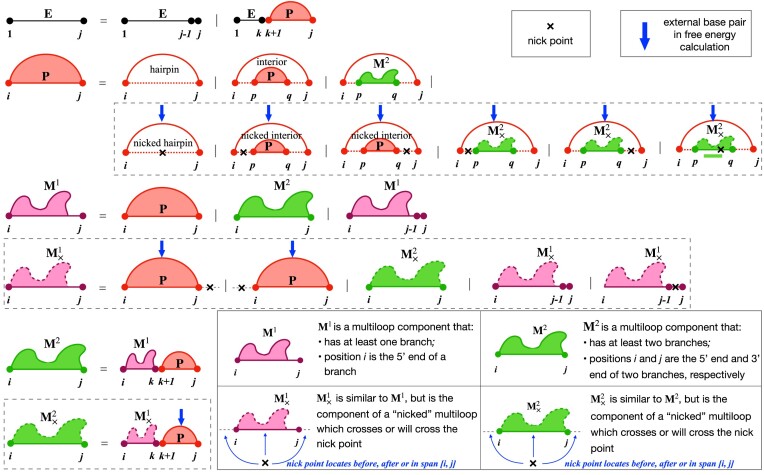
Deductive system of LinearCoFold and LinearCoPartition based on the Zuker system. For single-strand folding (ignoring dashed boxes), four states **E**(*i*, *j*), **P**(*i*, *j*), **M**^1^(*i*, *j*) and **M**^2^(*i*, *j*) are defined to retain the MFE structure for the span [*i*, *j*], where **P**(*i*, *j*) requires *i* paired with *j*, **M**^1^(*i*, *j*) and **M**^2^(*i*, *j*) are the components of multiloops. To extend to two-strands cofolding (adding dashed), first, LinearCoFold takes into consideration the nicked hairpin, nicked interior loop and nicked multiloop for state **P**(*i*, *j*). Besides, LinearCoFold adds two states $\mathbf {M}^{1}_{\times }(i, j)$ and $\mathbf {M}^{2}_{\times }(i, j)$ to model the components of nicked multiloops. More importantly, the innermost base pairs enclosing the nick point to form **P**(*i*, *j*) (first dashed box), as well as the closing base pairs of branches of **P**(*i*, *j*) when forming $\mathbf {M}^{1}_{\times }(i, j)$ and $\mathbf {M}^{2}_{\times }(i, j)$ (second and third dashed boxes) are treated as external base pairs since the nick point makes the loop exterior. The blue arrows indicate external base pairs due to the existing of nick point. Note that LinearCoFold only picks up the MFE structure, while LinearCoPartition sums up all possible structures for each state.

Compared to the Nussinov–Jacobson energy model, the Zuker system based on the Turner energy mode defines more states to represent different types of loops. Formally, for single-strand folding, state **E**(*i*, *j*), **P**(*i*, *j*), **M**^1^(*i*, *j*) and **M**^2^(*i*, *j*) retain the MFE structure for the span [*i*, *j*], where **P**(*i*, *j*) requires *i* paired with *j*, **M**^1^(*i*, *j*) has at least one branch with *i* as the 5’ end of the leftmost branch, and **M**^2^(*i*, *j*) contains at least two branches with *i* and *j* as the 5’ end and the 3’ end of the leftmost and rightmost branches, respectively (Figure 4 except for dashed boxes). Extending to two-strand cofolding, LinearCoFold takes into consideration of the nicked hairpin, nicked interior loop and nicked multiloop for state **P**(*i*, *j*), and also adds two states $\mathbf {M}^{1}_{\times }(i, j)$ and $\mathbf {M}^{2}_{\times }(i, j)$ to model the components of nicked multiloops; shown in dashed boxes in Figure 4. Note that the base pairs pointed by the blue arrows in Figure 4 are treated as external base pairs in the free energy calculation, since they are external base pairs due to the existing of nick point. The intermolecular initiation free energy, $\Delta G^{\circ }_{\text{DuplexInit}}$, is added to the innermost base pair across the nick point.

### LinearCoPartition algorithm

Beyond the MFE structure, a partition function and base-pairing probabilities of cofolding two RNA strands, and their assembled structure from the ensemble (e.g., MEA structure) are desired in many cases. A partition function *Q*(**x**) sums the equilibrium constants of all possible secondary structures in the ensemble. Using the revised Nussinov-Jacobson energy model, the partition function of two interacting RNAs can be formalized as:


(4) \begin{eqnarray*} && Q(\mathbf {x}) = \sum _{\mathbf {y} \in \mathcal {Y(\mathbf {x})}} e^{-\frac{\Delta G^{\circ }_{\mathbf {w}}({\mathbf {x}, \mathbf {y}})}{RT}}\nonumber\\ && \quad = \sum _{\mathbf {y} \in \mathcal {Y(\mathbf {x})}} \ \ \bigl ( \prod _{\substack{k \in {\rm {unpaired}}(\mathbf {y})}} e^{-\frac{\delta (\mathbf {x}, k)}{RT}} \cdot \prod _{\substack{(i,j) \in {\rm {pairs}}(\mathbf {y})}} e^{-\frac{\phi (\mathbf {x}, \mathbf {y}, i, j)}{RT}}\bigl ) \end{eqnarray*}

where *R* is the universal gas constant and *T* is the absolute temperature, and $\phi (\mathbf {x}, \mathbf {y}, i, j)$ is defined in Equation (2).

We further extend LinearCoFold to LinearCoPartition based on the inside-outside algorithm following LinearPartition (29), calculating the local partition function *Q*_*i*, *j*_ in a left-to-right order. Supplementary Figures S2 and S3 shows simplified pseudocode based on the Nussinov-Jacobson model. LinearCoPartition consists of two major steps: partition function (‘inside phase’) and base-pairing probability (‘outside phase’) calculations, where the outside phase is symmetrical to the inside phase but in a ‘right-to-left’ order. The base-pairing probability *p*_*i*, *j*_ can be derived if position *i* can be paired with *j*.

### Distinguishability and order of concatenation

Dirks *et al.* discussed the distinguishability issue of multi-strand folding, i.e. some of the permutations of strands are indistinguishable due to physical symmetries (27), which need to be ruled out. In the case of two-strand folding, however, there are only two indistinguishable permutations: *AB* or *BA*, where *A* and *B* are the two RNA strands. Therefore, in standard two-strand folding systems such as RNAcofold, only one of the two permutations needs to be considered, i.e., the predictions are the same when switching the two input sequences. But in LinearCoFold and LinearCoPartition, the beam pruning heuristic may prune out different states when switching the input sequences, resulting in different predictions. We notice that LinearCoFold and LinearCoPartition have higher accuracy on benchmark dataset when using an oligo-first order (i.e. shorter sequence as the first input sequence and the longer one as the second). Therefore, we place the shorter strand in front of the longer one by default, and provide the options for users to run in a customized order.

It is worth mentioning that Dirks *et al.* also discussed the distinguishability issue for identical input sequences, which results in algorithmic overcounting in partition function even when ruling out indistinguishable permutations. In our case, we assume the input sequences are different.

## RESULTS

### Datasets

We compared the performance of LinearCoFold and LinearCoPartition to RNAcofold on two datasets. The first dataset, collected by Lai and Meyer (19), contains 109 pairs of bacterial sRNA-mRNA sequences and 52 pairs of fungal snoRNA-rRNA sequences with annotated ground truth of intermolecular base pairs. The sRNA-mRNA interactions are originally from CopraRNA (45), among which 18 conserved enterobacterial sRNAs and 82 verified mRNA targets are curated by Lai and Meyer; the interactions are experimentally validated by the introduction of compensatory mutations in sRNA and target. The snoRNA–rRNA interactions are originally from the Methylation Guide snoRNA Database (46) and the UMASS Amherst Yeast snoRNA Database (47), which include 43 snoRNAs and 2 rRNAs. The combined sequence length in this dataset ranges from 546 *nt* to 3651 *nt*. We refer to this dataset as the Meyer dataset, and use it for the efficiency and accuracy benchmarks. The second dataset contains 16 miRNA-mRNA pairs from the TargetScan database (48). We first sampled 16 mRNA sequences ranging from 2411 to 100 275 *nt*, and sampled 16 miRNA sequences ranging from 15 *nt* to 28 *nt*, and then randomly assemble them into 16 miRNA-mRNA pairs with combined sequence length (i.e. *n* + *m*) ranging from 2432 to 100 297 *nt*. We refer this dataset as the TargetScan dataset in the paper, and use it for the efficiency benchmark only. For benchmarks, we used a Linux machine (CentOS 7.9.2009) with 2.40 GHz Intel Xeon E5-2630 v3 CPU and 16 GB memory, and gcc 4.8.5. We used the default setting of RNAcofold; the beam size *b* of LinearCoFold and LinearCoPartition is set to 100 (default value) for all experiments. Note that the default value of *b* is inherited from LinearFold (28), which shows that the performance of *b* = 100 is robust across different families.

### Efficiency and scalability

**Figure 5.**
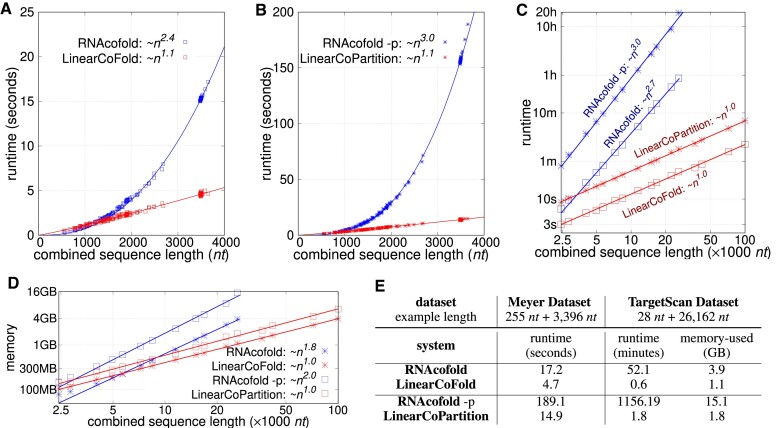
Runtime and Memory usage comparisons between RNAcofold and our algorithms. (A, B) Runtime against sequence length on the Meyer dataset; RNAcofold (MFE mode) and LinearCoFold are compared in (**A**), while RNAcofold-p (partition function mode) and LinearCoPartition are compared in (**B**). (**C**) runtime against sequence length on the TargetScan dataset. (**D**) memory usage against sequence length on the TargetScan dataset. Note that (C) and (D) are plotting in the log-log scale. (**E**) the performance comparisons on two selected examples from the two dataset. The example from the Meyer dataset is one of the sequences that have the longest combined length, and the example from the TargetScan dataset is the longest one that RNAcofold can run. The experiments were run on a Linux machine (CentOS 7.9.2009, 2.40 GHz Intel Xeon E5-2630 v3 CPU and 16 GB memory); the compiler is gcc 4.8.5.

We first investigated the efficiency of LinearCoFold and LinearCoPartition by plotting the runtime against the combined sequence length, and compared them to Vienna RNAcofold on the Meyer dataset, whose sequences are relatively shorter than the TargetScan dataset. Figure 5A and B clearly shows that our LinearCoFold and LinearCoPartition both achieve linear runtime with the combined sequence length; in contrast, RNAcofold runs in nearly cubic time (MFE mode, Figure 5A) or exactly cubic time (partition-function mode, Figure 5B) in practice. Our algorithms are substantially faster than RNAcofold on long sequences (*n* + *m* > 1500 *nt*). For one of the longest combined sequences with length of 3651 (255+3396) *nt*, LinearCoFold is 3.7× faster than RNAcofold MFE mode (4.7 s versus 17.2 s), and LinearCoPartition is 12.7× faster than RNAcofold partition-function mode (14.9 s versus 189.1 s).

Figure 5C presents the efficiency and scalability comparisons on the TargetScan dataset in log-log scale. The two blue lines illustrate that RNAcofold’s runtime scales (close to) cubically on the long sequences, and the two red lines confirm that the runtime of LinearCoFold and LinearCoPartition are indeed linear. We also observed that LinearCoFold and LinearCoPartition can scale to sequences of length 100 000 *nt* in 2.2 and 6.9 min, respectively, while RNAcofold cannot process any sequences with combined sequence length longer than 32 767 *nt*.

For the longest sequence pair (combined sequence length 26 190 *nt*) in the dataset that RNAcofold can run, LinearCoFold is 86.8× faster than RNAcofold MFE mode (0.6 min versus 52.1 min), and surprisingly, LinearCoPartition is 642.3× faster than RNAcofold partition-function mode (1.8 min versus 1156.2 min).

The memory usage on the TargetScan dataset is shown in Figure 5D. From the plots in log–log scale, we can see that the memory required by our LinearCoFold and LinearCoPartition increases linearly with the sequence length, while it scales quadratically for RNAcofold. For the longest one within the scope of RNAcofold, LinearCoFold takes 28.2% of memory compared to RNAcofold MFE mode (1.1 GB versus 3.9 GB), and LinearCoPartition takes only 11.9% of memory compared to RNAcofold partition-function mode (1.8 GB versus 15.1 GB).

### Accuracy

We compared the accuracy of LinearCoFold and LinearCoPartition to RNAcofold on the Meyer dataset. Due to the absence of the annotation of intramolecular base pairs in the Meyer dataset, the accuracy evaluation is limited to intermolecular pairs. More specifically, we removed all intramolecular base pairs from the prediction, and calculated Positive Predictive Value (PPV, the fraction of predicted pairs in the annotated base pairs) and sensitivity (the fraction of annotated pairs predicted) to measure the accuracy only for intermolecular base pairs across the two families in the Meyer dataset, and got the overall accuracy averaged on the two families.

**Figure 6.**
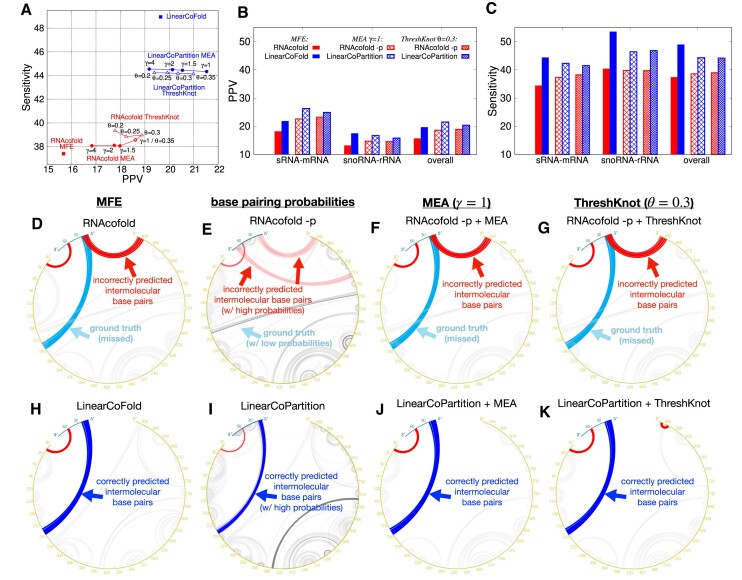
Prediction accuracy comparison between Vienna RNAcofold and our algorithms on the Meyer dataset. (**A**) PPV against sensitivity of the MFE structures (RNAcofold 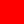 versus LinearCoFold 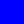, the MEA structures with varying *γ* of 1, 1.5, 2 and 4 (RNAcofold 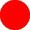 versus LinearCoPartition 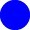), and the ThreshKnot structures with varying *θ* of 0.2, 0.25, 0.3 and 0.35 (RNAcofold 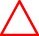 vs. LinearCoPartition 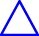). (**B**) and (**C**) per family and overall PPV and sensitivity comparisons between the six systems; we choose *γ* = 1 for MEA and *θ* = 0.3 for ThreshKnot since they are the default values. Note that all the experiments are performed in the default order, i.e., short sequence first; we also present the results of the reverse order in Supplementary Figure S5. (**D–K**) circular plots of the MFE structure, the base pair probabilities, the MEA structure (*γ* = 1) and the ThreshKnot structure (*θ* = 0.3) generated from RNAcofold (D–G) and ours (H–K) on a bacterial sRNA–mRNA sequence pair (MG1655 and NC_000913.3), respectively; each arc represents a base pair (the darkness of the arc represents the pairing probability in E and I). The cyan arcs are the ground truth intermolecular base pairs; the blue arcs are the correct predictions and the red arcs are the incorrect predictions. The intramolecular base pairs are colored in gray.

Figure 6A shows the overall PPV and sensitivity on the Meyer dataset. Compared to RNAcofold MFE mode, the overall PPV and sensitivity of LinearCoFold increase 4.0% and 11.6%, respectively. For the MEA structure prediction, we plotted a curve with varying *γ* (a parameter balances PPV and sensitivity in the MEA algorithm) from 1 to 4; compared to RNAcofold MEA, LinearCoFold MEA shifts to the top-right corner, which means that it has higher PPV and sensitivity. For *γ* = 1, the overall PPV and sensitivity of LinearCoPartition MEA increase 2.9% and 5.7%, respectively. In addition, for the ThreshKnot structures (38), we plotted a curve with varying *θ* (a parameter balances PPV and sensitivity in the ThreshKnot algorithm) from 0.2 to 0.35; compared to RNAcofold ThreshKnot, LinearCoFold ThreshKnot also shifts to the top-right corner. For *θ* = 0.3, the overall PPV and Sensitivity of LinearCoPartition ThreshKnot increase 1.5% and 5.2%, respectively. Figure 6B, C and Supplementary Figure S4 show the PPV, sensitivity and Matthews Correlation Coefficient (MCC) (49,50) comparisons on each family, respectively, confirming that LinearCoFold and LinearCoPartition are more accurate than RNAcofold on both bacterial sRNA–mRNA and fungal snoRNA–rRNA families. We observed that both RNAcofold’s and our predictions have higher PPV but lower sensitivity on sRNA-mRNA, and no substantial differences in MCC between the two types. Notably, MCC of LinearCoFold is lower than LinearCoPartition on sRNA-mRNA family, but higher on snoRNA–rRNA family. This is likely due to the fact that mRNA sequences normally have multiple conformations, and LinearCoPartition, as a partition function-based system which considers competing alternative structures (29), is more principled for mRNA-related structure prediction.

On a bacterial sRNA-mRNA sequence pair (OmrA sRNA, 88 *nt*; csgD mRNA, 951 *nt*), we illustrated the MFE structures, the base-pairing probabilities, the MEA structures (*γ* = 1) and the ThreshKnot structures (*θ* = 0.3) generated from RNAcofold MFE mode, partition-function (-p) mode, as well as LinearCoFold and LinearCoPartition (Figure 6D–K). Each arc in the circular plots represents a base pair. The darkness of the arc represents its probability in the base-pairing matrix (Figure 6E and I). The intramolecular base pairs are in gray, while the intermolecular base pairs are marked using different colors to represent the correctly predicted pairs (blue), the ground-truth pairs but missing in the prediction (cyan), and the incorrectly predicted pairs (red). We observed that all of our predictions correctly detect the intermolecular base pairs between 5’-end of the first strand and around 230 *nt* of the second strand (blue arcs in Figure 6H–K), while all of RNAcofold structures do not have these interactions (cyan arcs in Figure 6D–G), also incorrectly predict interactions between 5’ end of the first strand and 3’ end of the second strand (red arcs in Figure 6D–G). It is worth mentioning that the higher sensitivity and PPV of LinearCoFold and LinearCoPartition are inherited from LinearFold and LinearPartition, which rule out structures with low probabilities that are less likely to be the ground truth structures (28,29).

### The prediction of host-virus RNA–RNA interaction

SARS-CoV-2 virus is likely to be a long-time threat to global health, and great effort was made to better understand the virus, including studies that modeled its structure (51–54).

**Figure 7.**
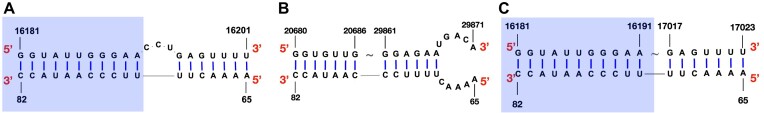
LinearCoFold’s prediction of the interaction between SARS-CoV-2 gRNA and human U4 snRNA better correlates with the wet lab experiments. (**A**) the structure of SARS-CoV-2 gRNA and human snRNA U4 interacting region detected by the wet lab experiment. (**B**) RNAcofold’s prediction of the interacting structure. (**C**) LinearCoFold’s prediction of the interacting structure. The blue rectangles highlight the region that LinearCoFold correlates with the wet lab experiment.

On the other hand, it is known that viral genomes interact with the host RNAs and form duplex structure, and predicting such host-virus RNA–RNA interaction is of great interest. A previous study (54) found that the SARS-CoV-2 genomic RNA (gRNA) binds with human U4 small nuclear RNAs (snRNAs), and illustrated their interacting structures, which are visualized in Figure 7A. We can see that the [65, 82] region of human U4 snRNA forms helices with the [16181, 16201] region of SARS-CoV-2 gRNA, and a 3-nucleotide bulge loop locates in the [16192, 16194] region. Figure 7B shows that the predicted structure from RNAcofold does not match the wet lab experiment results, in which the [70, 82] region of human U4 snRNA pairs with the downstream region of SARS-CoV-2 gRNA. By contrast, LinearCoFold’s prediction, shown in Figure 7C, has intermolecular base pairs between the [73, 82] region of human U4 snRNA and the [16181, 16191] region of SARS-CoV-2 gRNA, which overlaps with the experimental results and correctly predicts 11 out of 18 intermolecular base pairs.

## DISCUSSION

### Summary

We present LinearCoFold and LinearCoPartition for the secondary structure prediction of two interacting RNA molecules. Our two algorithms follow the strategy used in RNAstructure bifold and Vienna RNAcofold, which concatenates two RNA sequences and distinguishes ‘normal loops’ from loops that contains nick point, to simplify two-strand folding into the classical single-strand folding, and predict both intramolecular and intermolecular interactions. Based on this, LinearCoFold and LinearCoPartition further apply beam pruning heuristics to reduce the cubic runtime in the classical RNA folding algorithms, resulting in a linear-time prediction of minimum free energy structure (LinearCoFold) and a linear-time computation of partition function and base pairing probabilities (LinearCoPartition). Unlike other *local* cofolding algorithms, LinearCoFold and LinearCoPartition are *global* linear-time algorithms, which means that they do not have any limitations of base pairing distance, allowing the prediction of global structures involving long distance interactions. We confirm that:

1. LinearCoFold and LinearCoPartition both run in linear time and space, and are orders of magnitude faster than Vienna RNAcofold. On a sequence pair with combined length of 26190 *nt*, LinearCoFold is 86.8 × faster than RNAcofold MFE mode, and LinearCoPartition is 642.3× faster than RNAcofold partition function mode. See Figure 5.
2. Evaluated on the Meyer dataset with annotated intermolecular base pairs, LinearCoFold and LinearCoPartition’s predictions have higher PPV and sensitivity. The overall PPV and Sensitivity of LinearCoFold increase +4.0% and +11.6% over RNAcofold MFE, respectively; LinearCoPartition MEA increases +2.9% on PPV and +5.7% on sensitivity over RNAcofold MEA, and LinearCoPartition TheshKnot increases +1.5% on PPV and +5.2% on sensitivity over RNAcofold TheshKnot. See Figure 6A–C. A case study on a bacterial sRNA-mRNA sequence pair is provided to show the difference of predicted structures. See Figure 6D–K.
3. LinearCoFold can predicts interaction between viral genomes and host RNAs. For the SARS-CoV-2 gRNA interacting with human U4 snRNA confirmed by a previous wet lab study, LinearCoFold correctly predicts 11 out of 18 intermolecular base pairs, while RNAcofold predicts 0 out of 18. See Figure 7.

### Extensions

Our algorithm has several potential extensions.

1. Multiple RNAs can form into complex conformations, but current algorithms and tools are built on the classical *O*(*n*^3^) folding algorithms, and are slow for long sequences (27). Our LinearCoFold and LinearCoPartition are extendable from two-strand cofolding to multi-strand folding.
2. Following LinearSampling (31), a linear-time stochastic sampling algorithm for single strand, our LinearCoPartition is extendable to LinearCoSampling for the sampling of the cofolding structures.

## Supplementary Material

gkad664_Supplemental_Files

## Data Availability

The data (used as efficiency and accuracy benchmarks) and code have been deposited at https://doi.org/10.5281/zenodo.8153422.
