# Supplementary material for: LinearCoFold and LinearCoPartition: linear-time algorithms for secondary structure prediction of interacting RNA molecules
Source: Nucleic Acids Res. 2023 Aug 31;51(18):e94. doi: 10.1093/nar/gkad664 (PMC10570024; doi:10.1093/nar/gkad664)
Supplement: gkad664_Supplemental_Files [file gkad664_supplemental_files.zip › si.pdf]

Supporting Information

LinearCoFold and LinearCoPartition: Linear-Time Secondary Structure Prediction Algorithms of Interacting RNA molecules

He Zhang, Sizhen Li, Ning Dai, Liang Zhang, David H. Mathews and Liang Huang

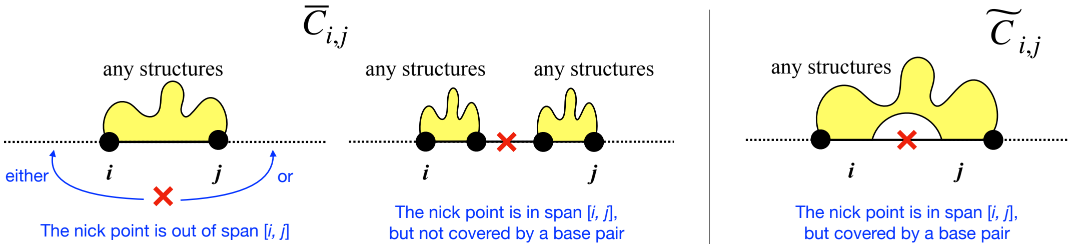

function LINEARCOFOLD($\mathbf{x}^a, \mathbf{x}^b, b$) ▷ b: beam size
  $n \leftarrow$ length of $\mathbf{x}^a$, $m \leftarrow$ length of $\mathbf{x}^b$ ▷ n and m: sequence lengths
  $\mathbf{x} \leftarrow \mathbf{x}^a \circ \mathbf{x}^b$ ▷ concatenate two sequences
  $\overline{C} \leftarrow$ hash(), $\widetilde{C} \leftarrow$ hash() ▷ hash tables: from span $[i,j]$ to $\overline{C}_{i,j}$ and $\widetilde{C}_{i,j}$
  $\overline{C}_{j,j-1} \leftarrow 0$ for all j in $1 \ldots n+m$ ▷ base cases
  for $j = 1 \ldots n+m$ **do**
    for each i such that $[i, j-1]$ in $\overline{C}$ **do** ▷ $O(b)$ iterations
      $\overline{C}_{i,j} \leftarrow \overline{C}_{i,j-1} + \delta(\mathbf{x}, j)$ ▷ skip
      if $x_{i-1}x_j$ in {AU, UA, CG, GC, GU, UG} **then**
        for each k such that $[k, i-2]$ in $\overline{C}$ **do** ▷ $O(b)$ iterations
          if $i-1 > n$ **or** $j \le n$ **then** ▷ not innermost intermolecular base pair
            $\overline{C}_{k,j} \leftarrow \min(\overline{C}_{k,j}, \overline{C}_{k,i-2} + \overline{C}_{i,j-1} + \xi(\mathbf{x}, i-1, j))$ ▷ pop
        if $i-1 \le n$ **and** $j > n$ **then** ▷ innermost intermolecular base pair
          $\widetilde{C}_{i-1,j} \leftarrow \min(\widetilde{C}_{i-1,j}, \overline{C}_{i,j-1} + \xi(\mathbf{x}, i-1, j)) + G^{\circ}_{\text{DuplexInit}}$ ▷ lift
        for each k such that $[k, i-2]$ in $\widetilde{C}$ **do** ▷ $O(b)$ iterations
          $\widetilde{C}_{k,j} \leftarrow \min(\widetilde{C}_{k,j}, \widetilde{C}_{k,i-2} + \overline{C}_{i,j-1} + \xi(\mathbf{x}, i-1, j))$ ▷ pop
    for each i such that $[i, j-1]$ in $\widetilde{C}$ **do** ▷ $O(b)$ iterations
      $\widetilde{C}_{i,j} \leftarrow \min(\widetilde{C}_{i,j}, \widetilde{C}_{i,j-1} + \delta(\mathbf{x}, j))$ ▷ skip
      if $x_{i-1}x_j$ in {AU, UA, CG, GC, GU, UG} **then**
        for each k such that $[k, i-2]$ in $\overline{C}$ **do** ▷ $O(b)$ iterations
          $\widetilde{C}_{k,j} \leftarrow \min(\widetilde{C}_{k,j}, \overline{C}_{k,i-2} + \widetilde{C}_{i,j-1} + \xi(\mathbf{x}, i-1, j))$ ▷ pop
    LINEARCOFOLDBEAMPRUNE($\overline{C}$, $\widetilde{C}$, j, b) ▷ choose top b out of $\overline{C}(\cdot, j)$
    LINEARCOFOLDBEAMPRUNE($\widetilde{C}$, $\overline{C}$, j, b) ▷ choose top b out of $\widetilde{C}(\cdot, j)$

function LINEARCOFOLDBEAMPRUNE(C', C, j, b)
  $candidates \leftarrow$ hash() ▷ hash table: from candidate i to score
  for each i such that $[i, j]$ in C' **do**
    $candidates[i] \leftarrow \min(C'_{1,i-1}, C_{1,i-1}) + C'_{i,j}$
  $candidates \leftarrow$ SELECTTOPB($candidates$, b) ▷ select top-b states by score
  for each i such that $[i, j]$ in C' **do**
    if key i not in $candidates$ **then**
      delete $[i, j]$ from C' ▷ prune low-scoring states

Figure SI 1. Pseudocode of a simplified version of the LinearCoFold. In the pseudocode, we use states $\overline{C}_{i,j}$ and $\widetilde{C}_{i,j}$ to store the best score of span $[i,j]$, where $\overline{C}_{i,j}$ is for spans without nick point or with uncovered nick point, and $\widetilde{C}_{i,j}$ is for spans with covered nick point; shown on the top of the figure. At each step j, three actions, SKIP, POP, and LIFT are performed. SKIP extends $\overline{C}_{i,j-1}$ (or $\widetilde{C}_{i,j-1}$) to $\overline{C}_{i,j}$ (or $\widetilde{C}_{i,j}$) by adding an unpaired base $\mathbf{y}_j =$ "$\cdot$" to the right of the span $[i, j-1]$. POP combines span $[i, j-1]$ with an upstream span $[k, i-2]$ and updates the resulting $\overline{C}_{k,j}$ (or $\widetilde{C}_{k,j}$) if x_{i-1} can be paired with x_j. LIFT is a special action converting $\overline{C}_{i,j-1}$ to $\widetilde{C}_{i-1,j}$ if (x_{i-1}, x_j) is the innermost intermolecular base pair, where the free energy change $\xi(\mathbf{x}, i, j) + G^{\circ}_{\text{DuplexInit}}$ is used. The real LinearCoFold system is more involved, but the pseudocode illustrates the left-to-right partition function calculation idea using a Nussinov-Jacobson model.

```
function LinearCoPartitionInside(x^a, x^b, b)                              ▷ b: beam size
    n ← length of x^a, m ← length of x^b                                   ▷ n and m: sequence lengths
    x ← x^a ∘ x^b                                                          ▷ concatenate two sequences
    Q̄ ← hash(), Q̃ ← hash()                                                 ▷ hash tables: from span [i,j] to Q̄_{i,j} and Q̃_{i,j}
    Q̄_{j,j−1} ← 1 for all j in 1...n+m                                     ▷ base cases
    for j = 1...n+m do
        for each i such that [i, j−1] in Q̄ do                              ▷ O(b) iterations
            Q̄_{i,j} += Q̄_{i,j−1} · e^{−δ(x,j)/RT}                          ▷ skip
            if x_{i−1}x_j in {AU, UA, CG, GC, GU, UG} then
                for each k such that [k, i−2] in Q̄ do                      ▷ O(b) iterations
                    if i−1 > n or j ≤ n then                               ▷ not innermost intermolecular base pair
                        Q̄_{k,j} += Q̄_{k,i−2} · Q̄_{i,j−1} · e^{−ξ(x,i−1,j)/RT}   ▷ pop
                if i−1 ≤ n and j > n then                                  ▷ innermost intermolecular base pair
                    Q̃_{i−1,j} += Q̄_{i,j−1} · e^{−(ξ(x,i−1,j)+G°_DuplexInit)/RT}  ▷ lift
                for each k such that [k, i−2] in Q̃ do                      ▷ O(b) iterations
                    Q̃_{k,j} += Q̃_{k,i−2} · Q̄_{i,j−1} · e^{−ξ(x,i−1,j)/RT}  ▷ pop
        for each i such that [i, j−1] in Q̃ do                              ▷ O(b) iterations
            Q̃_{i,j} += Q̃_{i,j−1} · e^{−δ(x,j)/RT}                          ▷ skip
            if x_{i−1}x_j in {AU, UA, CG, GC, GU, UG} then
                for each k such that [k, i−2] in Q̄ do                      ▷ O(b) iterations
                    Q̃_{k,j} += Q̄_{k,i−2} · Q̃_{i,j−1} · e^{−ξ(x,i−1,j)/RT}  ▷ pop
        LinearCoPartitionBeamprune(Q̄, Q̃, j, b)                             ▷ choose top b out of Q̄(·, j)
        LinearCoPartitionBeamprune(Q̃, Q̄, j, b)                             ▷ choose top b out of Q̃(·, j)

function LinearCoPartitionBeamprune(Q′, Q, j, b)
    candidates ← hash()                                                    ▷ hash table: from candidates i to score
    for each i such that [i, j] in Q′ do
        candidates[i] ← (Q′_{1,i−1} + Q_{1,i−1}) · Q′_{i,j}                ▷ use Q_{1,i−1} as prefix score
    candidates ← SelectTopB(candidates, b)                                 ▷ select top-b states by score
    for each i such that [i, j] in Q′ do
        if key i not in candidates then
            delete [i, j] from Q′                                          ▷ prune low-scoring states
```

Figure SI 2. Pseudocode of a simplified version of the LinearCoPartition, including partition function calculation (inside phase).

function LINEARCOPARTITIONOUTSIDE($\mathbf{x}^a, \mathbf{x}^b, b, \overline{Q}, \widetilde{Q}$) ▷ outside calculation
  $n \leftarrow$ length of $\mathbf{x}^a$, $m \leftarrow$ length of $\mathbf{x}^b$ ▷ n and m: sequence lengths
  $\mathbf{x} \leftarrow \mathbf{x}^a \circ \mathbf{x}^b$ ▷ concatenate two sequences
  $\widehat{\overline{Q}} \leftarrow$ hash(), $\widehat{\widetilde{Q}} \leftarrow$ hash() ▷ hash table: from span $[i,j]$ to $\widehat{\overline{Q}}_{i,j}$ and $\widehat{\widetilde{Q}}_{i,j}$
  $p \leftarrow$ hash() ▷ hash table: from span $[i,j]$ to $p_{i,j}$: base-pairing probability
  $\widehat{\overline{Q}}_{1,n+m} \leftarrow \widetilde{Q}_{1,n+m}$ ▷ base case
  $\widehat{\widetilde{Q}}_{1,n+m} \leftarrow \overline{Q}_{1,n+m}$ ▷ base case
  for $j = n+m$ **down to** 1 **do**
    for each i such that $[i, j-1]$ in $\widetilde{Q}$ **do** ▷ $O(b)$ iterations
      $\widehat{\widetilde{Q}}_{i,j-1} \mathrel{+}= \widehat{\widetilde{Q}}_{i,j} \cdot e^{-\frac{\delta(\mathbf{x},j)}{RT}}$ ▷ skip
      if $x_{i-1}x_j$ in {AU, UA, CG, GC, GU, UG} **then**
        for each k such that $[k, i-2]$ in $\overline{Q}$ **do** ▷ $O(b)$ iterations
          $\widehat{\overline{Q}}_{k,i-2} \mathrel{+}= \widehat{\widetilde{Q}}_{k,j} \cdot \widetilde{Q}_{i,j-1} \cdot e^{-\frac{\xi(\mathbf{x},i-1,j)}{RT}}$ ▷ pop
          $\widehat{\widetilde{Q}}_{i,j-1} \mathrel{+}= \widehat{\widetilde{Q}}_{k,j} \cdot \overline{Q}_{k,i-2} \cdot e^{-\frac{\xi(\mathbf{x},i-1,j)}{RT}}$ ▷ pop
          $p_{i-1,j} \mathrel{+}= \frac{\widehat{\widetilde{Q}}_{k,j} \cdot \overline{Q}_{k,i-2} \cdot \widetilde{Q}_{i,j-1}}{Q_{1,n+m}}$ ▷ accumulate base pairing probs
    for each i such that $[i, j-1]$ in $\overline{Q}$ **do** ▷ $O(b)$ iterations
      $\widehat{\overline{Q}}_{i,j-1} \mathrel{+}= \widehat{\overline{Q}}_{i,j} \cdot e^{-\frac{\delta(\mathbf{x},j)}{RT}}$ ▷ skip
      if $x_{i-1}x_j$ in {AU, UA, CG, GC, GU, UG} **then**
        for each k such that $[k, i-2]$ in $\overline{Q}$ **do** ▷ $O(b)$ iterations
          if $i-1 > n$ **or** $j \leq n$ **then** ▷ not innermost intermolecular base pair
            $\widehat{\overline{Q}}_{k,i-2} \mathrel{+}= \widehat{\overline{Q}}_{k,j} \cdot \overline{Q}_{i,j-1} \cdot e^{-\frac{\xi(\mathbf{x},i-1,j)}{RT}}$ ▷ pop
            $\widehat{\overline{Q}}_{i,j-1} \mathrel{+}= \widehat{\overline{Q}}_{k,j} \cdot \overline{Q}_{k,i-2} \cdot e^{-\frac{\xi(\mathbf{x},i-1,j)}{RT}}$ ▷ pop
            $p_{i-1,j} \mathrel{+}= \frac{\widehat{\overline{Q}}_{k,j} \cdot \overline{Q}_{k,i-2} \cdot \overline{Q}_{i,j-1}}{Q_{1,n+m}}$ ▷ accumulate base pairing probs
        if $i-1 \leq n$ **and** $j > n$ **then** ▷ innermost intermolecular base pair
          $\widehat{\widetilde{Q}}_{i,j-1} \mathrel{+}= \widehat{\widetilde{Q}}_{i-1,j} \cdot e^{-\frac{\xi(\mathbf{x},i-1,j)+G^{\circ}_{\text{DuplexInit}}}{RT}}$ ▷ lift
          $p_{i-1,j} \mathrel{+}= \frac{\widehat{\widetilde{Q}}_{i-1,j} \cdot \overline{Q}_{i,j-1}}{Q_{1,n+m}}$ ▷ accumulate base pairing probs
        for each k such that $[k, i-2]$ in $\widetilde{Q}$ **do** ▷ $O(b)$ iterations
          $\widehat{\widetilde{Q}}_{k,i-2} \mathrel{+}= \widehat{\widetilde{Q}}_{k,j} \cdot \overline{Q}_{i,j-1} \cdot e^{-\frac{\xi(\mathbf{x},i-1,j)}{RT}}$ ▷ pop
          $\widehat{\widetilde{Q}}_{i,j-1} \mathrel{+}= \widehat{\widetilde{Q}}_{k,j} \cdot \widetilde{Q}_{k,i-2} \cdot e^{-\frac{\xi(\mathbf{x},i-1,j)}{RT}}$ ▷ pop
          $p_{i-1,j} \mathrel{+}= \frac{\widehat{\widetilde{Q}}_{k,j} \cdot \widetilde{Q}_{k,i-2} \cdot \overline{Q}_{i,j-1}}{Q_{1,n+m}}$ ▷ accumulate base pairing probs
  return p ▷ return the (sparse) base-pairing probability matrix

Figure SI 3. Pseudocode of a simplified version of the LinearCoPartition, including base pairing probability calculation (outsite phase).

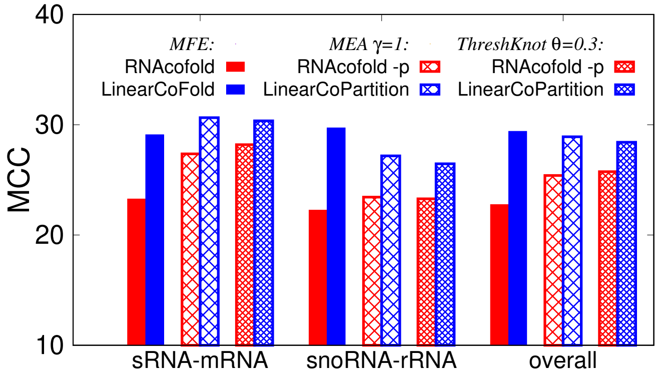

Figure SI 4. The Matthews Correlation Coefficient (MCC) of LinearCoFold's and LinearCoPartition's predictions compared with RNAcofold's, benchmarked on the Meyer's dataset.

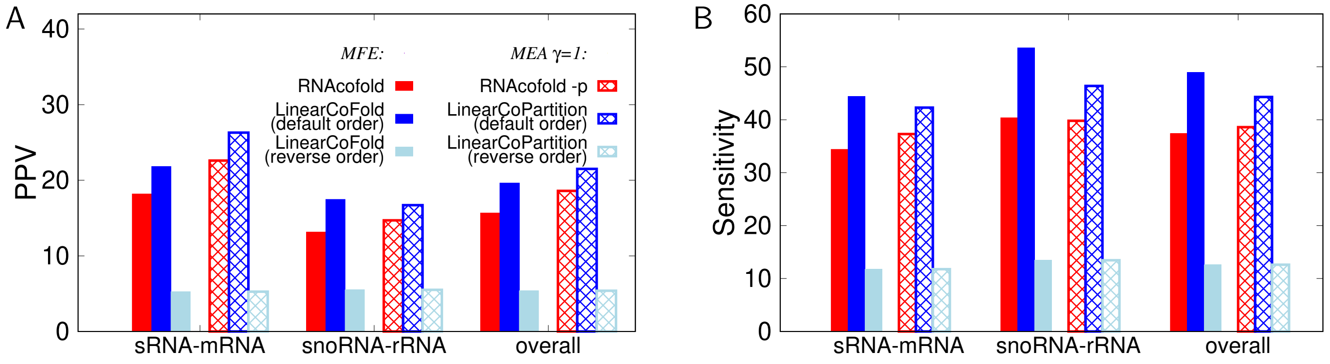

Figure SI 5. The accuracies of LinearCoFold and LinearCoPartition drop when reversing the default order of the two input sequences, i.e., longer sequence as the first input sequence and shorter sequence as the second one in the reverse order.
